# Investigating the psychosocial determinants of physical activity in older adults: A qualitative approach

**DOI:** 10.1080/08870446.2016.1143943

**Published:** 2016-03-10

**Authors:** Maria-Christina Kosteli, Sarah E. Williams, Jennifer Cumming

**Affiliations:** ^a^ School of Sport, Exercise and Rehabilitation Sciences, University of Birmingham, Birmingham, UK

**Keywords:** social cognitive theory, self-efficacy, barriers, enablers, retirement

## Abstract

***Objective:*** Despite the benefits of physical activity (PA), only one-third of older adults meet the recommended levels. The present study focused on psychosocial determinants of PA following retirement. Social cognitive theory (SCT) was used to better understand pre- and post-retirement adults’ thoughts about PA, the reasons why some individuals are more active than others, and how PA is incorporated into daily life after retirement.

***Design:*** Seven focus groups of older adults (*N* = 37, *M* = 64, SD = 5.20; males = 20) representing a range of PA levels and retirement length participated in one of seven focus groups.

***Results:*** Aligned with SCT, self-efficacy beliefs along with perceptions about barriers and benefits of PA were among the major determinants of PA. Findings highlighted the importance of social support, positive outcome expectations and self-regulatory strategies as motivators. The lack of structure in retirement was a hindrance to incorporating PA into daily routine but, when incorporated, PA provided a sense of purpose in the lives of retired individuals.

***Conclusion:*** It is important to understand the meaning of retirement as a life transition and how it affects beliefs about PA to inform SCT-based health promotion interventions targeting individuals in retirement age.

Physical activity (PA) contributes to healthy ageing through prevention of chronic age-associated illnesses and disabilities (Peel, McClure, & Bartlett, 2005). The biological and psychological benefits of PA include, but are not limited to, reduced risk of cardiovascular disease, hypertension, osteoporosis and depression (Taylor et al., 2004). Despite the many benefits, the majority of older adults do not engage in regular PA or exercise (Prohaska et al., 2006). The Department of Health (2013) recommends that older adults participate in moderate PA for at least 150 min a week, but only 58% of men and 52% of women between 65 and 74 years achieve these guidelines. Percentages can be even lower in times of transition such as retirement (Laverty & Flint, 2014), making this a particularly important stage of life to investigate.

Retirement can be experienced either as a beginning of a new phase in life or as an imposed disruption of life patterns (Hornstein & Wapner, 1985). Because of the numerous changes associated with this transition (e.g. decreased income, reduced social contact, loss of daily routine), retirement can be important in determining PA behaviour. Despite retired individuals seemingly having more free time to incorporate PA into their lives, a systematic review of 19 quantitative studies and 5 qualitative studies indicated that while recreational PA can increase, overall PA generally decreases following retirement (Barnett, Ogilvie, & Guell, 2011). However, there is still equivocal evidence for the impact of retirement on overall PA levels (Barnett, van Sluijs, & Ogilvie, 2012). Factors thought to influence lower PA during retirement include loss of daily structure, lack of exercise partners, adjustment to retirement patterns and financial constraints (McDonald, O’Brien, White, & Sniehotta, 2015).

In the process of understanding the PA behaviour of individuals entering retirement, the relevant psychosocial factors must also be determined. Social cognitive theory [SCT]; Bandura, 1997) has been shown to explain 52% of exercise behaviour in post-retired individuals (Schuster, Petosa, & Petosa, 1995). The theory refers to the societal influences on human thought and behaviour, and the subsequent impact of these thoughts on one’s motivation, attitudes, and action (Stajkovic & Luthans, 1998). SCT proposes a set of psychosocial constructs (i.e. self-efficacy, outcome expectations, barriers and self-regulatory strategies) that determine health behaviours such as PA.

A major premise of SCT is that belief in one’s ability to successfully perform the desired behaviour (i.e. self-efficacy) is a primary determinant of the adoption of PA (Bandura, 1997), particularly in older adults (Conn, 1998). Of the different types of self-efficacy (e.g. task efficacy), the majority of older adult PA research (e.g. McAuley, Jerome, Marquez, Elavsky, & Blissmer, 2003) has focused on barrier efficacy (i.e. belief in one’s capability to engage in PA under challenging conditions such as bad weather and boredom; Bandura, 1997). Irrespective of self-efficacy type, individuals with higher self-efficacy are more likely to engage in the PA behaviour, put forth more effort and persist in the face of difficulties (Bandura, 1986). Therefore, it is a key construct to target when developing PA interventions.

The second important variable in SCT is outcome expectations, defined as ‘the perception that a certain behaviour will bring out specific outcomes’ (Bandura, 1977; p.193). Individuals are more likely to engage in PA when they perceive these efforts will lead to important positive outcomes such as better health (Resnick, 2001). People who expect positive health benefits as a result of PA are more likely to set goals and plans related to PA (Ayotte, Margrett, & Hicks-Patrick, 2010). However, it is also important to account for the degree to which these outcome expectations are met over time (Wilcox, Castro, & King, 2006).

Regardless of an individual’s beliefs in the benefits of PA, real and perceived barriers to PA can determine whether or not individuals engage in PA (Bandura, 1997). Barriers can be internal (e.g. lack of motivation) or external (e.g. adverse weather conditions). Health concerns are one of the most frequently reported barriers to exercise in older adults (Crombie et al., 2004), and the more perceived barriers to PA for an individual, the less likely they are to regulate their behaviour to engage in PA (Ayotte et al., 2010).

Self-regulation strategies enable individuals to cope with perceived barriers to PA and include goal setting, planning, problem solving and self-monitoring (Bandura, 1997). Self-regulation is among the most influential social-cognitive variables for changing health behaviour because it guides behaviour towards a directed aim or goal (Anderson, Wojcik, Winett, & Williams, 2006). Thus, it can play a key role in the adoption of PA in older adults with more frequent use of self-regulatory strategies relating to greater self-efficacy and PA engagement (Umstattd, Wilcox, Saunders, Watkins, & Dowda, 2008). SCT proposes that self-efficacy acts through self-regulation to influence behaviour (Bandura, 1986). Indeed, previous research with older adults indicates that self-regulation mediates the relationship between self-efficacy and PA (Umstattd et al., 2008). However, few studies have examined the role of self-regulation strategies in helping adults to be physically active post-retirement. Better understanding of the relationship between self-regulation and other SCT constructs may prove helpful in explaining PA behaviour in older adults.

Despite the aforementioned associations between SCT constructs, these relationships are far from simple and the different constructs can interact in complex ways. Ayotte et al. (2010) examined several predictions of SCT with older adults and found that higher self-efficacy both directly and indirectly related to greater PA through fewer perceived barriers, more positive outcome expectancies and higher self-regulatory behaviour. These findings demonstrate the importance of including all these constructs to more fully understand the PA patterns of older adults.

Most studies investigating how SCT relates to the PA patterns of older adults have used a quantitative approach. To gain a more complete picture, a qualitative approach would further our understanding of how social-cognitive factors act to promote and impede PA in retirement. Understanding how older adults experience retirement, and perceive PA, may shed light on the reasons behind variations in post-retirement activity levels. This understanding could then inform the development of theory-based interventions to help older adults overcome the barriers associated with PA.

Using focus groups, the purpose of the present study was to gain a more in-depth understanding of the reasons and motives for why some retired individuals are sufficiently active, whereas others are insufficiently active. To date, only six qualitative studies have examined PA in retirement (for a review, see Barnett et al., 2011; also see McDonald et al., 2015). Unlike these previous qualitative studies, the present one was guided by SCT. To gain a range of perspectives, another novel aspect of the study was the inclusion of both pre-retired and retired individuals of varying lengths who also varied in their PA behaviour.

## Method

### Participants

Thirty-seven (male = 20) older adults from West Midlands, UK between 54 and 79 years old (*M* = 64, SD = 5.20) were invited to participate in focus groups arranged by self-reported levels of PA and retirement length. Twenty-six participants reported engaging in moderate/vigorous PA for more than 2.5 h per week, while 11 reported being insufficiently active due to engaging in less than 2.5 h of PA per week (Department of Health, 2013). Eighteen participants reported retiring between 2 and 5 years ago, six retired more than 10 years ago, and thirteen either planned to retire within the next two to three years or were within their first year of retirement. Most participants (*n* = 36) were of white British origin, and one was of mixed origin.

### Procedure

Following ethical approval, participants were recruited from the local community through flyers and were invited to participate in one of seven focus groups. Purposeful sampling was used to recruit individuals varying in PA levels and retirement length to allow for a range of different perspectives to emerge in the discussions. Sampling reached completion when data saturation was achieved (Francis et al., 2010). Thus, the participants were classified in one of the following distinct focus groups: active and 1st year retired, insufficiently active and 1st year retired, active and retired for 2–5 years, insufficiently active and retired for 2–5 years, active and retired for longer than 10 years. These relatively homogenous focus groups were formed with the aim of providing a trusting environment composed of ‘similar others’. Such an environment would allow opinions to unfold freely through the interaction of participants with similar characteristics. As explained by Smithson (2000), the interactive nature of the focus groups is a major benefit of qualitative research. Focus groups ranged in size from 3 to 6 individuals. The primary author served as the focus group moderator and led the discussion, while an observer took notes and kept track of the time. Each discussion was audio recorded and lasted between 48 and 89 min.

Participants were informed about the purpose of the study and how confidentiality would be maintained. They then provided written consent by completing a consent form and provided their age, gender, employment status, ethnicity and PA level. Participants were then informed about the format of the group discussion and asked to introduce themselves and tell a little about themselves such as when they retired, and the way they travelled to the university. These introductory questions were used to establish trust and rapport with the participants (Stewart & Shamdasani, 2014).

### Interview guide

Focus groups followed a semi-structured interview guide, initially pilot tested with a group of researchers experienced in qualitative and/or older adult research. The interview guide included open-ended questions, drawn from SCT, related to participants’ perceptions about PA. Questions and included the benefits, thoughts and feelings, barriers, enablers of PA and participant’s confidence they will become or remain active after retirement. Questions were adapted to be relevant for the different PA levels and retirement length focus groups. As participants shared their experiences, probes were used to retrieve more in-depth information about their thoughts and feelings.

### Analysis

Focus groups were transcribed verbatim resulting in 340 pages of double-spaced transcripts. Data were analysed using thematic analysis (Frith & Gleeson, 2004) aligned to the critical realist and post-positivist standards of rigour, which recognises that accounts from participants are subjective and represent their own lived ‘reality’, holding a personal meaning for them, influenced by sociocultural factors (Willig, 1999). Based on the recommendations of Braun and Clarke (2006), the following steps were followed during the thematic analysis: (1) data familiarisation; (2) initial coding; (3) sort codes into themes; (4) refine themes; (5) define and label the themes; (6) devil’s advocate meeting; and (7) thematic structure finalised.

Initial codes were generated with an open coding process (Strauss & Corbin, 2008). An inductive analysis allowed for exploration of unanticipated findings not accounted for in previous research, which might not have otherwise emerged using a deductive approach to examine the data within SCT (Braun & Clarke, 2006). The themes were reviewed and further organised in hierarchical structures called categories (Strauss & Corbin, 2008). Data were reanalysed with a deductive approach to determine the utility of SCT to explain PA pre- and post-retirement. This ensured a better understanding of the emergent themes based on SCT and checked if any themes could be generalised (Burns & Grove, 2005). Second, comparisons were made between the inductively derived data and deductively created categories. Themes emerging inductively that did not fit the social-cognitive framework were retained if they were considered to be important to the description of the phenomenon.

### Issues of trustworthiness

To establish an accurate, valid and reliable representation of the participants’ PA perceptions, several procedures were followed. First, the authors had regular meetings to discuss the findings throughout the data analysis. During the initial stages, multiple coders compared codes and reached an agreement. In later stages, the interpretation of findings was compared among the researchers until a consensus was reached. This was to challenge the interpretation of the overall findings and serve as a method of triangulation of evidence by multiple investigators (Creswell & Miller, 2000). Second, the higher order themes were presented to a group of researchers unaffiliated with the present study. In these meetings, a ‘devil’s advocate’ approach was followed (MacDougall & Baum, 1997). Random quotes representing each higher ordered theme were presented to the group and agreement among the members was established.

Thick description (Tracy, 2010) also established trustworthiness. This included providing a very detailed description of the participants, environment and specific situation. A better understanding of the context enables readers to make judgements regarding the applicability of the findings. Researcher reflexivity was another strategy used to ensure credibility and allowed the researchers to express their beliefs and biases (Thomas & Magilvy, 2011). For this purpose, a self-reflective journal was kept where the primary researcher expressed her feelings, biases and assumptions she made during this research. Discussions with the other authors made sure that the researchers’ assumptions were not reflected on the interpretations. Finally, extensive quotes from the participants were included in the results section as a way to let readers judge the accuracy of the authors’ conclusions.

## Results

Participants were offered a broad definition of PA, which allowed for a wide range of perceptions of PA to emerge. Specifically, PA was defined as any structured activity with the goal of fitness and health, but it also involves other lifestyle activities. Some participants associated PA with playing sports or going to the gym, whereas others referred to walking, gardening and housework. Thus, the personal meaning of PA varied and influenced perceptions of how physically active they considered themselves to be. The analysis identified three higher order themes associated with PA: (1) determinants of PA; (2) outcomes of PA; and (3) perceived barriers and enablers. Quotations from the participants are presented to illustrate themes and subthemes and ensure that findings represent the participants’ voices. Activity level, gender and retirement length are provided to give context to each quote.

### Determinants of PA

Determinants of PA referred to the motives and reasons why older adults engaged in PA. Five subthemes emerged: (1) physical well-being; (2) psychological well-being; (3) socialising; (4) health perceptions; and (5) physical demand of previous occupation.

#### Physical well-being

Older adults referred to exercising for the purpose of remaining healthy and in a good physical condition. Health was the most cited reason for engaging in PA among participants. PA was either prescribed to them by a physician or was identified as necessary by the participants themselves. As described by one participant**,** ‘For health reasons it’s been advised I did Pilates for my back’ (Active female/2–5 years retired). Similarly, a recently retired individual said, ‘Walking is good for the heart, blood pressure, and cholesterol levels’ (Active female/1st year retired).

#### Psychological well-being

Older adults regularly and frequently talked about experiencing positive psychological outcomes or avoiding negative psychological outcomes as a result of being active. Generally, the participants described four reasons for engaging in PA: enjoyment, mood regulation, relaxation and purpose in life.

#### Enjoyment

Most participants referred to pleasure as the main reason for engaging in PA. For example, ‘Well I do walking, not fast walking; just moderate walking just because I enjoy it that’s it, it’s not because I think of any depth benefit, I just enjoy it’ (Active female/2–5 years retired). Enjoyment was equally important to individuals who were about to retire. For instance, a participant reported, ‘ If I’m out in the rain I don’t mind. The weather doesn’t really bother me. So with me it’s mainly enjoyment from actually doing whatever I’m doing’ (Active male/pre-retiree). However, unenjoyable exercise activity could lead to a lack of motivation as explained by another participant, ‘I don’t go to the gym because I don’t enjoy gyms’ (Active male/2–5 years retired).

#### Mood regulation

Avoiding negative psychological outcomes such as depression was frequently highlighted as a reason for PA participation. One participant said, ‘I think I would be quite depressed if I didn’t do physical exercise of some manner’ (Active male/1st year retired). However, PA was also a way for older adults to generally feel better and experience feelings of happiness. As explained by one participant, ‘If I’m not active psychologically it affects me and it makes me feel that I ought to be out doing something. I don’t feel as well as if I have been out doing physical activities’ (Active male/2–5 years retired).

#### Relaxation

Experiencing a feeling of calm was a commonly identified reason for PA engagement, as shown by the following statement, ‘After doing a good walk for about an hour or two I feel a lot looser in my body and more relaxed after it’ (Active female/pre-retiree).

#### Purpose in life

A few participants reported that PA gave them something to look forward to following retirement along the lines of, ‘I think even more so now that I’m retired and you need purpose in your life and you think yes I’ve done something worthwhile and so on’ (Active female/Retired for 2–5 years).

#### Socialising

Most participants acknowledged PA was a key part of their social life, such as to meet and interact with people. The social side of PA seems to be as important as the PA for older adults. One participant stated, ‘So that’s why I do it, it’s the social effect of meeting people’ (Active female/>10 years retired).

#### Health perceptions

Some participants talked about being satisfied with their health and thus not finding PA necessary. In other words, holding positive health perceptions could deter older adults from engaging in PA. Having a reason to engage in PA appeared to be an important PA determinant for older adults, ‘If it’s for a purpose I’m fine, but I don’t see a point in going to a gym for the sake of it, if you’re fit and healthy anyway’ (Insufficiently active male/2–5 years retired).

#### Physical demand of previous occupation

A final factor that determined PA levels in older adults was the physically demanding nature of their previous occupation. Some participants who retired from sedentary occupations reported being more physically active in retirement as a result of previously having jobs that did not require a high level of PA. For example, a participant said, ‘I’m a lot more active since I retired because my job was sitting in an office easy access to facilities’ (Active male/2–5 years retired). However, other participants described being less physically active in retirement as a result of retiring from an occupation that involved high levels of PA. One insufficiently active participant explained,

I was very active at work. I was a removal man for 10 years. Then I got off the road and into sewer maintenance. So I was always active. When I retired I think I just couldn’t be bothered to get off my ass. (Insufficiently active male/2–5 years retired)

Recently retired individuals also expressed lower PA levels due to physically demanding occupation. One individual reported, ‘I was a carer, so that’s PA. If you work for 40 h doing it and you’re not working now, you do a lot less now’ (Active female/1st year retired).

### Outcomes of PA

The outcomes of PA referred to the benefits older adults experienced as a direct result of being active. Two themes emerged that were termed: (1) physical outcomes; and (2) psychological outcomes.

#### Physical outcomes

There were very few physical outcomes mentioned by participants, and of these, it all pertained to a good night’s sleep.

#### Good night’s sleep

A few of the participants referred to sleeping better as a result of PA. For example, a participant said, ‘The day I did yoga I slept much more relaxed than the days I didn’t do yoga’ (Active female/2–5 years retired).

#### Psychological outcomes

Psychological outcomes referred to by a few participants were related to good mood and a sense of achievement/satisfaction.

#### Good mood

Most participants mentioned being in a good mood and experiencing positive feelings as a result of PA. According to a recently retired participant, ‘It’s a happy mood you would be in yes; feeling a lot better that you did than dossing around doing nothing’ (Active female/1st year retired).

#### Sense of achievement/satisfaction

Many participants referred to a feeling of accomplishment when completing a physically demanding task. Feeling able to do what they wanted to do seemed to be an important outcome of PA. Older adults described PA as a rewarding experience that makes them feel satisfied once they overcome the challenge and accomplish their goal. For example,

It’s so nice to feel that you’ve still got that ability to do that and I think that’s partly why I keep doing it for the satisfaction of getting that feeling of pleasure from the fact I can still run round for a couple of hours. (Active female/2–5 years retired)

The same was the case for individuals who had not yet retired. For instance, an individual admitted, ‘There’s satisfaction in having achieved something and those achievements can be self-set targets within the target if you like. So you can create your own levels of satisfaction’ (Active male/pre-retiree).

### Barriers

Barriers described factors that could limit PA engagement. Two categories emerged in this theme: (1) personal; and (2) social-environmental barriers.

#### Personal barriers

Personal barriers were factors related to participants such as lack of time, pain, lack of motivation, health perceptions, lack of structure/routine and dislike for the exercise setting.

#### Lack of time

Lack of time could be either a real or perceived barrier that could limit PA participation. Some older adults were not motivated to adopt PA in their lifestyles and thus they perceived having limited amount of time after retirement. A participant stated, ‘My barrier is making time to do it. I’m not really stretching my time since I finished work’ (Insufficiently active male/2–5 years retired). However, lack of time could also reflect a real obstacle that some participants faced. For example, one of the participants reported having family or personal commitments that prevented him from exercising, along the lines of ‘Hang on, people are pulling me this way, that way and perhaps it should be that everybody should designate some of my time only for me’ (Active male/2–5 years retired). Similarly, some individuals reported that lack of time was their motive for retiring, along the lines of, ‘Time was my problem, I never had time because I was working. One reason I’m retiring is because I want more time to be able to do activity’ (Insufficiently active/pre-retiree).

#### Pain

Aches and pains associated with PA reduced its enjoyment and prevented people from being active. A few participants referred to pain as a warning sign and something that they should avoid as highlighted by one of the oldest participants, ‘Muscles sort of hurting during exercise, which as you say can be a warning that perhaps you shouldn’t push it any further’ (Active female/>10 years retired). The idea of avoiding pain in older age was also highlighted by one of the younger participants who was about to retire. He said: ‘As you get older you’re told as soon as you get anything, any ache or pain you should stop and that’s what I’ve tended to do’ (Active male/pre-retiree). However, pain perception varied due to the individual’s PA level. More active people were more familiar with stretching themselves and perceived it as normal,

I think if you’ve done PA throughout your life you are used to that, you are used to pushing yourself a little bit. If you wake up the following day and you’re a bit stiff you know why it is and you just get over it. (Active male/2–5 years retired)

#### Lack of motivation

Some participants talked about lacking motivation to initiate PA. This was mostly the case for those who reported being insufficiently active.

I know I need to lose weight. PA would probably help. As I say I know what I should do, I know how I should do it, it’s not a problem it’s just getting the motivation to do it. You know I’d rather sit down with a book and a chocolate bar than go out and run three miles these days. (Insufficiently active male/1st year retired)

Lack of motivation was also a concern for insufficiently active participants who had been retired longer. One participant explained, ‘I think basically I’m just lazy, nothing to do so I don’t do anything’ (Insufficiently active male/2–5 years retired).

#### Lack of structure

Some participants particularly those who were insufficiently active discussed the impact of having less structure post-retirement. This could be defined as the lack of routine or simply disorganisation that comes from the abundance of free time as explained by one participant,

I think most of us have said it would be nice if we didn’t have to work or that sort of thing. Now we’ve got it, it’s more difficult to organise, because we are in total charge. Nobody tells us what to do or guides us, we have to work it out for ourselves. (Insufficiently active male/2–5 years retired)

Lack of structure also resonated with more active people who were about to retire. For example, a participant said ‘I think you do less when you’ve got more time than when you’ve actually got to do it in an hour, you actually get it done whereas you can put off doing things’ (Active female/pre-retiree). The lack of structure seemed to have a great effect on recently retired individuals as well. Another recently retired participant said, ‘I find not so much gets done as it did before because in a routine you have to do it, now it’s oh shall we go out, shall we leave it, lets get up later … this is all new’ (Active female/1st year retired)

#### Social comparison

Another barrier discouraging older adults to engage in PA was comparisons they made between themselves to others who are younger or more fit. A participant reported,

It would stop me going to a gym because you see all these fit young people who are slim and not overweight. So you tend to think well it’s for younger people, it’s not for me. You see in magazines and its young people running and they’re offering sports equipment or sports drinks and you rarely see anybody over 30 in those adverts. (Insufficiently active male/1st year retired)

#### Social-environmental barriers

Social-environmental barriers involved external influences that affected people’s involvement in PA such as adverse weather conditions, lack of exercise partner and financial constraints.

#### Adverse weather conditions

Regardless of PA level and the stage of retirement, the majority of participants talked about being discouraged to exercise in the presence of inclement weather such as rainy or snowy or short days. The weather severity influenced whether people were active along the lines of, ‘I do pick my days. Like yesterday there was a chance of a shower but I went and I took my umbrella. But if I knew it was going to rain I wouldn’t go in the rain’ (Active male/2–5 years retired). Individuals about to retire also found the weather off-putting and expressed the same idea along the lines of, ‘In the winter when it’s really coming down or it’s really bad winter like snow, it does put you off’ (Active male/pre-retiree).

#### Lack of exercise partner

Several older adults perceived lack of an exercise partner to be a barrier when it comes to exercise. For example, ‘For me personally I like somebody to do it with and I find that difficult’ (Insufficiently active female/1st year retired). Another recently retired individual found that being away from the work setting minimised the opportunities for PA. He said,

I’m doing less now because I don’t have the range of people to ask for a game or things to do. The sort of ability to walk across the corridor and say do you fancy a game just isn’t there. It’s much more organised. It has to be. (Active male/1st year retired)

#### Financial constraints

Some participants perceived they couldn’t afford to participate in certain types of PA because there is a decrease in income as a result of retirement. For these participants, PA was associated with having a gym membership or participating in organised sport activities that required membership fees or other incurred expenses. One participant said, ‘Obviously retired I’ve got less money than when I was at work so you know perhaps some more exciting activities you know you can’t do’ (Active female/2–5 years retired). Similarly, a recently retired individual reported, ‘I think money is also an aspect now that I’m retired, I’ve got far less money, it’s more of a consideration now than it used to be’ (Active female/1st year retired).

### Enablers

This final theme included factors that could positively influence PA behaviours and encourage people to be active. Two categories emerged in this theme: (1) internal; and (2) external enablers.

#### Internal enablers

Internal enablers refer to factors under a person’s control that can motivate older adults to engage in PA. These involve time availability in retirement, self-regulation strategy and enjoyment of the PA setting.

#### Time availability in retirement

Most participants anticipated having more free time to exercise after retirement. For example, a recently retired participant stated, ‘Well I am retired so I have all the time in the world’ (Active female/1st year retired). Although participants acknowledged that retirement is associated with increased leisure time and more opportunities to become more physically active, not all utilised this time for PA purposes. Participants, who had been retired for a longer time, were aware of the challenges of having so much freedom, especially in the first few months after retirement. For example, a participant said,

I think the first 12 to 18 months after retirement are quite busy. You do a lot of things that you haven’t had time to do before like catching up with people, dashing here or there. After that initial period I think it just tires off and you gradually slow down. (Insufficiently active male/2–5 years retired)

#### Self-regulation strategy

A few participants talked about the importance of committing to an exercise routine either by setting a specific time for PA, and/or making plans to engage in PA with other people. One participant reported, ‘Commitment, in other words joining something or not letting other people down if you don’t go’ (Active female/2–5 years retired). Time off from PA could demotivate older adults getting back into it along the lines of, ‘I think having stopped then it’s harder to get started again’ (Insufficiently active male/1st year retired). To self-regulate their behaviour, older adults sometimes tried to find alternative ways to maintain PA. For instance, a participant said,

If you’ve got the option of a lift or the stairs try and use the stairs that sort of thing, as I do brisk walking, do a bit more of that or as you say walk to en extra bus stop. (Insufficiently active male/>10 years retired)

#### Enjoyment of PA setting

Many participants talked about enjoying and being positively influenced by the natural surroundings and location of the PA. The enjoyment and pleasure derived from the environment seemed to be more important than the activity itself and helped older adults overcome barriers like bad weather. For example, a participant said,

When I first started walking years ago I was a fair weather walker, I just walked during the good weather but now I walk right through the year and its lovely, you see nature and I really enjoy that, the birds and you see the winter and everything about it and those are the things that I enjoy. (Active female/2–5 years retired)

The exercise location and the positive feelings associated with it had a major impact on the participants’ motivation to engage in PA and facilitated engagement in PA. One participant explained, ‘I don’t like going to the gym and I find it hard to make the time to do that because I don’t want to do it’ (Active male/>10 years retired).

#### External enablers

External enablers refer to social and environmental factors that are somewhat beyond an individual’s control, and include good weather, having an exercise partner, and positive vicarious experience of PA.

#### Good weather

Across the sample, most of the older adults recognised the importance of good weather as a determinant factor in their engagement of PA. Lack of rain and warm weather were among the most important factors that determined whether older adults would engage in PA. A participant commented, ‘It’s much better in the summer than in the winter for walking or any outdoor activity, its far more enjoyable so that does play a part in it actually the weather and outside influences’ (Active female/pre-retiree).

#### Having an exercise partner

One of the most frequently reported sources of social support that motivated older adults to engage in PA was the presence of an exercise partner. Almost all participants reported the importance of having an exercise companion or being part of a group. An exercise partner could help older adults not only withstand the possible physical unpleasantness associated with PA but also motivate them to initiate the activity. For instance, a participant said, ‘If I’ve got a friend who went to the gym locally perhaps I might be more motivated’ (Active female/2–5 years retired). Similarly, insufficiently active older adults reported that doing PA with somebody else could motivate them to overcome barriers associated with it. A participant explained,

I got to get to the swimming pool, it’s awful, it’s windy, it’s raining, but if my lady friend goes with me she says get going and we do and you come back out of it and you do, well I certainly feel better. (Insufficiently active male/2–5 years retired)

The same positive effect could be achieved through having a non-human exercise partner, such as a dog. Specifically a participant reported,

I think if I wasn’t involved with walking the dog then perhaps I would degenerate into lazing around at home. I’m a dog walker for the past two years and when we had the dog to start with, it rained every single morning that I took the dog out and I thought I don’t really fancy it today but the dog needs its exercise. So you put on all your wet water equipment on and you just go and when the wind and the rain hits you, you think oh I … but you’re doing it for a purpose. (Active male/>10 years retired)

*Positive vicarious experience of PA*. Some participants described being inspired by other people’s experiences with PA. Seeing others engage in PA and the health benefits experienced from this was a strong motivator for older adults. A participant said, ‘I’ve had that example of people remaining fit in older, older age and I sort of think in myself … to keep as active as I can within the parameters I can do’ (Active female/2–5 years retired).

## Discussion

The goal of the present study was to investigate how pre- and post-retirees representing a variety of activity levels perceive PA and how they self-regulate their behaviour. The present study is one of the few studies that have focused specifically on the transition to retirement (McDonald et al., 2015), and the first to adopt Bandura’s SCT as a theoretical framework to understand the psychosocial determinants of PA behaviour during this transition. The variability in PA levels and retirement lengths allowed for a range of perceptions to emerge and shed light on the factors that influence PA during transition to retirement.

Findings are consistent with SCT and highlight self-efficacy as one of the underlying reasons for engaging in PA in individuals who are in retirement age. The participants reflected on whether they could retain a physically active lifestyle in retirement and identified several reasons that influenced their confidence levels. Specifically, findings highlighted that subjective health status, and time perception were related to their belief in their ability to remain active throughout retirement. Thus, perceived poor health and lack of time made them less confident that they could sustain PA in retirement. Furthermore, self-regulation was identified as another major reason for engaging in PA. Participants referred to strategies they used to regulate their behaviour such as paying gym subscription, getting an exercise partner and joining an exercise group. This supports previous research that indicates self-regulation as a key social-cognitive factor for changing health behaviour in older adults (Umstattd et al., 2008)

Overall, physically active older adults were more confident in their ability to maintain PA throughout retirement, expected fewer barriers, and perceived more benefits from exercising compared to insufficiently active individuals. Their beliefs in their capabilities to overcome certain barriers along with having positive outcome expectations appeared to facilitate their motives to initiate a PA regime. Although all participants held positive physical, psychological and social outcome expectations, only a few engaged in PA, while others remained insufficiently active. This supports previous research, which has shown that outcome expectations alone is not a strong predictor of PA (Rovniak, Anderson, Winett, & Stephens, 2002), whereas targeting self-efficacy can be an effective way to help older people overcome barriers to PA (Lee, Arthur, & Avis, 2008). Thus, boosting older adults’ confidence in their ability to sustain PA throughout retirement should be one of the primary aims of PA interventions for older adults.

In our study, the attitudes and beliefs that retirees had about the benefits and barriers of PA seemed to differ between activity levels. Active older adults perceived fewer barriers and more enablers to PA compared to those who were not sufficiently active, defined as exercising less than 2.5 h per week. Active older adults may have more resources to cope with the barriers they face in regards to PA. Furthermore, barriers seemed to differ across activity levels. Sufficiently active older adults reported more social-environmental barriers (e.g. adverse weather conditions), whereas less active individuals faced more personal barriers related to motivation (e.g. lack of organisation). Indeed, lack of motivation was the most frequently reported barrier for insufficiently active older adults.

Some barriers in the present study were consistent with previous research such as not having sufficient time to be physically active (Sherwood & Jeffery, 2000). Contrary to the common belief that retirement is associated with more free time and thus provides more opportunities for PA, it is important to note that a number of retired individuals perceive lack of time to be a barrier to PA – particularly those who are insufficiently active. Most physically active participants recognised that having time in retirement can facilitate engagement in PA. It is likely that PA is not a high priority for insufficiently active individuals and this is why they perceive having limited time in retirement. This supports research suggesting that lack of time can instead reflect lack of motivation towards PA (Bowles, Morrow, Leonard, Hawkins, & Couzelis, 2002). Thus, insufficiently active older adults likely use lack of time to rationalise their low PA levels. Accounting for PA level and exploring the attitudes and feelings older adults have towards PA can shed light on whether some barriers are real or just perceived.

All retired adults reported not having a daily routine to be a major barrier preventing the development of goals and plans for PA. Feeling in ‘charge of yourself’ was a new experience for newly retired adults, which made them less ready to incorporate PA into their daily routine. Particularly in the case of new retirees who have yet to establish a routine, it is likely that they feel less confident in their ability to incorporate PA in their daily lifestyle. This supports research suggesting that lack of structure in retirees’ daily lives can be a challenge (Pettican & Prior, 2011). Consequently, it is important to help older adults establish a new routine in retirement, through incorporating PA in their daily schedules.

Another commonly reported barrier by insufficiently active people was social comparison. Insufficiently active people compared themselves to younger and fitter people, which discouraged them from initiating PA. This contradicts social comparison theory, which suggests people compare themselves with others who have similarities on a particular domain (Festinger, 1954). The more similar people are to reference comparison groups, the more likely they will evaluate themselves through the comparisons. Although social comparisons are not always healthy, under certain circumstances they can facilitate engagement in PA. For instance, physically active people reflected on experiences of people who remained active until an old age and experienced positive health outcomes. Thus, instead of comparing themselves to younger and fitter people they were getting encouraged to participate in PA by following the example of people who had been physically active in the past.

Consistent with SCT, social comparisons in the form of positive vicarious experiences of PA seemed to be an important source of self-efficacy for older adults (Bandura, 1986). Vicarious experience or modelling refers to observing the consequences of another person’s actions and adjusting behaviour accordingly. Having examples of others who were physically active in an older age, helped individuals feel more confident that they too could remain active and experience similar positive outcomes as a result of PA. Booth, Owen, Bauman, Clavisi, and Leslie (2000) demonstrated that older adults who interacted with physically active individuals in their social circle tended to be more physically active themselves, indicating the importance of social modelling. Thus, PA behaviour change appears to occur through these observations and vicarious experiences (Bandura & Huston, 1961).

Despite the numerous barriers to PA, participants reported experiencing a number of motivators similar to those identified in previous research (Beck, Gillison, & Standage, 2010). The results of our study supported previously reported factors underpinning older adults’ PA motivation during the retirement transition. For instance, similar to Costello, Kafchinski, Vrazel, and Sullivan’s (2011) study, older adults reported that socialising is a big part of their PA. Furthermore, an exercise partner or belonging to a group positively influenced older adults to engage in PA, which is consistent with SCT and supports the importance of social support as a motivator for PA (Burke, Carron, Eys, Ntoumanis, & Estabrooks, 2006). However, exercising with people of similar age is considered more beneficial (Beauchamp, Carron, McCutcheon, & Harper, 2007), whereas exercising with older or younger people could result in negative social comparisons that hinder an individual engaging in PA. Thus, placing older adults in exercise groups with similar others seems to be an important goal for future interventions. Consistent with Peel, Douglas, Parry, and Lawton (2010), walking with a dog is another form of companionship that can give a sense of purpose to older adults and can motivate them to stay physically active.

In the present study, older adults who were confident in their ability to sustain PA throughout retirement were more likely to self-regulate their behaviour and enjoy PA. Both active and inactive retirees seemed to pick activities they enjoyed and felt comfortable doing. The majority of physically active participants reported engaging in PA because they enjoyed it rather than the health benefits associated with PA. This supports the importance of enjoyment, which is one of the strongest predictors of PA (Deci & Ryan, 2000). Moreover, positive feelings experienced during exercise can predict future behaviour (Rhodes & Kates, 2015) and enjoyment is likely to determine not only whether older adults engage in PA but also the type of activities chosen.

As well as enjoyment, the feeling of accomplishment and achievement after engaging in PA seemed to be important – particularly for older adults retired for more than 10 years. These individuals use PA as a mechanism to cope with ageing. The sense of accomplishment is the most important source of self-efficacy (Bandura, 1997). Although there is no previous research on the effect of performance accomplishments on older adults, the present findings suggest older adults who have previous positive experiences and success are going to be more confident in their ability to engage in PA. Helping older adults to focus on what their bodies can do rather than their physical limitations can be very self-fulfilling.

A limitation of the present study is that PA levels were self-reported, which can be subject to memory bias, and participants may have over- or underestimated their PA levels (Slootmaker, Schuit, Chinapaw, Seidell, & van Mechelen, 2009). Future research may wish to use more objective measures, such as accelerometers, to classify whether older adults meet the recommended levels of PA. Another limitation is that we do not know whether participants changed their PA habits throughout retirement. Future longitudinal research could follow a set of participants throughout different stages of retirement and see how PA may change. Future research could also differentiate between determinants of PA and exercise, and find differences and similarities in the type of barriers and enablers people face in the different activity categories. It could be that those who define PA as going to the gym face different set of determinants than those who define it as a lifestyle activity.

## Conclusions

In conclusion, the current study generated knowledge that can be used as the basis for interventions attempting to promote PA in older adults. Boosting self-efficacy, promoting enjoyable activities, creating similar-age exercise groups, positive reinforcement and improving time-management skills seem viable ways to help individuals in transition to retirement to become more active. The barriers physically active older adults face appear less under their control, while the barriers insufficiently active older adults face seem to be related to motivation and self-regulation. Enablers to PA for this population included commitment to an exercise group or class and the continuation of regular exercise. By understanding the unique challenges and facilitators to PA that older adults experience in retirement, interventions can be tailored to the population to maximise their effectiveness.

## Funding

This research was supported by a scholarship from the Economic and Social Research Council (ESRC) to the first author.

## Disclosure statement

No potential conflict of interest was reported by the authors.
